# Cardiac Arrest Survivors’ Perspectives to Inform the Co-Design of a Web-Based Support and Learning Platform: Qualitative Content Analysis

**DOI:** 10.2196/84432

**Published:** 2026-05-22

**Authors:** Annette Waldemar, Anders Bremer, Johan Israelsson, Katarina Heimburg, Per Nordberg, Erik Blennow Nordström, Kristofer Årestedt, Ingela Thylén

**Affiliations:** 1 Department of Health, Medicine and Caring Sciences, Linköping University, Linköping, Sweden; 2 Clinical Department of Cardiology, Vrinnevi Hospital, Norrköping, Region Östergötland, SE-601 83, Sweden, + 46 70 9690977; 3 Department of Health and Caring Sciences, Faculty of Health and Life Sciences, Linnaeus University, Växjö, Sweden; 4 Department of Internal Medicine, Kalmar County Hospital, Kalmar, Region Kalmar, Sweden; 5 Neurology, Department of Clinical Sciences, Lund University, Lund, Sweden; 6 Department of Cardiology, Skåne University Hospital, Lund, Sweden; 7 Department of Physiology and Pharmacology, Karolinska Institutet, Stockholm, Sweden; 8 Department of Clinical Sciences and Education, Center for Resuscitation Science, Karolinska Institutet, Stockholm, Sweden; 9 Department of Rehabilitation Medicine, Skåne University Hospital, Lund, Sweden; 10 Clinical Department of Cardiology, Linköping University Hospital, Linköping, Region Östergötland, Sweden

**Keywords:** cardiac arrest, co-design, cocreation, digital health intervention, focus groups, internet-based intervention, patient participation, patient preference, qualitative research, self-management support, user-centered design

## Abstract

**Background:**

Survivors of cardiac arrest often face multifaceted challenges—cognitive, emotional, physical, and existential—that extend beyond clinical recovery. Despite these long-term consequences, follow-up care is often insufficient, and access to reliable information and support remains limited. Broader initiatives to address post–cardiac arrest care are still lacking. This qualitative study represents the initial phase of a multiphase development process to cocreate, design, and later evaluate a web-based support and learning platform for cardiac arrest survivors. The platform is intended to complement existing health care services and support survivors in managing life after cardiac arrest.

**Objective:**

This study aimed to explore survivors’ perspectives on digital support and identify relevant content and delivery formats for a web-based support and learning platform.

**Methods:**

Eight women and 12 men (aged 44‐80 years) were recruited via a moderated peer support network for cardiac arrest survivors. Time since cardiac arrest ranged from 3 months to 19 years. Data were collected between November 2024 and February 2025 through 3 individual and 4 focus group interviews, analyzed using qualitative content analysis.

**Results:**

Three main categories—(1) *digital communication and guided health care navigation*, (2) *digital opportunities to support recovery and address unmet needs*, and (3) *digital and interpersonal pathways to safe social contexts*—were identified as key design requirements for digital support. Substantial gaps in post–cardiac arrest care emerged, including fragmented and sometimes contradictory information, regional disparities, and limited psychosocial follow-up, underscoring the value of a national, accessible, trustworthy web-based program that complements standard care throughout recovery. Flexible formats—such as short videos, read-aloud functions, and information available both as concise and more in-depth versions—were considered essential to accommodate fatigue and cognitive difficulties. A digital platform was further identified as uniquely suited to gather relevant information in one place, provide expert-based explanations and links to further resources, and offer practical tools that could be accessed at home. Across categories, several unmet needs emerged as particularly suited to digital delivery, including guidance on health and everyday decisions, support for managing emotional and physical aftermath, resources to navigate altered social relations, intimacy and personality changes, and dedicated support for family members, who often lack tailored and continuous follow-up.

**Conclusions:**

Findings underscore the need for a tailored digital support program that extends beyond clinical encounters and offers structured, accessible, and personalized guidance across the recovery trajectory. By addressing long-term cognitive, physical, emotional, and relational needs, a contextually adapted digital program has the potential to bridge existing gaps in post–cardiac arrest care and strengthen survivors’ recovery. These user-driven insights provide a foundation for the cocreation and iterative development of a clinically grounded and adaptable digital support platform.

## Introduction

Many survivors of cardiac arrest experience cognitive, emotional, physical, and existential challenges that affect their everyday lives [1-4]. These difficulties often manifest as symptoms of anxiety, depression, posttraumatic stress, fatigue [5], sleep disturbances [6,7], and kinesiophobia [8], with fatigue and sleep problems frequently persisting long after the cardiac arrest event [9]. A recent state-of-the-art review highlights that psychological distress after cardiac arrest may also involve heightened vigilance to bodily signals, behavioral avoidance, and physiological hyperarousal, all of which can hinder recovery and increase long-term health risks [10].

The transition from hospital to home is often described as abrupt and disorienting [11,12], and many survivors report a lack of structured follow-up and adequate support to manage life after the event [13]. A poor understanding of the arrest and its consequences can contribute to uncertainty and distress [14]. Evidence from broader cardiac populations suggests that patients who feel unprepared for discharge may be at higher risk of readmission [15], although similar data specific to cardiac arrest survivors are lacking. Conversely, patients who feel well informed report an increased sense of control and a reduced need for emergency care [11]. Despite survivors’ desire to discuss the event and receive an early follow-up at cardiac clinics [11,16], many are left without such opportunities [17].

International and national postresuscitation care guidelines emphasize the importance of follow-up within 3 months, including patient education and psychosocial screening [18,19]. However, care after cardiac arrest is often fragmented and unevenly distributed across health care levels and regions [20]. Survivors—particularly those with non–cardiac arrest etiology—may fall outside the scope of specialist clinics and are often left to seek support through primary care or digital platforms [17]. As a result, many turn to online and community-based resources to find knowledge, connection, and tools for self-management [21].

Recent work has highlighted that community-based and nongovernmental organizations play an important role in addressing the diverse informational, emotional, social, and practical needs of cardiac arrest survivors—needs that often extend beyond formal health care services. A recent exploratory survey conducted by the European Resuscitation Council identified only a small number of organizations worldwide that provide structured and continuous support for survivors, including peer support networks and information platforms such as Sudden Cardiac Arrest UK and Heartsight in the United States [21]. These organizations vary widely in their activities and in the extent to which they are connected to national health care systems, with many reporting limited or no formal integration. Although some centers refer patients to these organizations at discharge, their overall integration into formal health care pathways remains limited, and they have not been scientifically evaluated as structured support interventions. Moreover, they are not available in Swedish or tailored to the Swedish health care context, where follow-up practices and access to services differ substantially. There remains a need for evidence-informed, contextually adapted digital support that is developed through a systematic exploration of survivors’ needs and cocreated with stakeholders within the national health care system.

Digital support interventions have been developed and evaluated in several other patient groups, including cancer, stroke, and heart failure, where web-based programs have shown potential to improve self-management, psychological well-being, and patient engagement [22-24]. Systematic reviews in these areas highlight the value of accessible, person-centered digital tools that combine education, peer support, and behavioral strategies [25,26]. Together, these findings indicate that digital support can be effective across a range of chronic and acute conditions, but they also highlight the importance of tailoring content and delivery to the specific needs of each survivor group. However, the needs of cardiac arrest survivors differ in important ways. Cognitive impairment, fatigue, memory difficulties, and posttraumatic stress are common [1,5] and may affect how survivors process information and engage with digital content. Moreover, unlike cancer or stroke, there is no established rehabilitation pathway or structured follow-up model for cardiac arrest survivors, and their access to care is often fragmented. These differences underscore the importance of developing support that is specifically tailored to this population and grounded in a systematic exploration of their lived experiences.

Digital solutions, grounded in a person-centered approach, could serve as an accessible means of delivering evidence-based education and psychosocial support regardless of diagnosis, geography, or health care structure [27]. However, to ensure that such interventions are meaningful and relevant, they must be cocreated with input from those who have experienced cardiac arrest.

There is currently limited knowledge about what survivors themselves perceive as relevant in terms of content, structure, and delivery of digital support. Understanding these preferences is a crucial first step in cocreating effective and tailored interventions. This qualitative study therefore forms part of a broader initiative to cocreate and evaluate a digital support program for cardiac arrest survivors, intended as a complement to existing health care services. The aim was to explore survivors’ perspectives on digital support and identify relevant content and delivery formats for a web-based support and learning platform.

## Methods

This study adheres to the COREQ (Consolidated Criteria for Reporting Qualitative Research) checklist [28].

### Study Design

A qualitative design was used, integrating focus group interviews and individual interviews to gain an in-depth understanding of survivors’ experiences and preferences regarding the content and delivery of potential support components. The work represents the initial phase of a multiphase development process aimed at cocreating, designing, and later evaluating a web-based support and learning platform for cardiac arrest survivors. The qualitative findings will directly inform the subsequent design and early development of the digital platform.

### Setting

The study was conducted in Sweden between November 2024 and February 2025. Participants were recruited via advertisements in the National Peer Support Network for Cardiac Arrest Survivors and Their Family Members. Additional advertisements were placed on the Swedish Heart and Lung Association’s national website and local bulletin boards. Survivors who expressed interest to participate contacted the research team via a dedicated study email address. Potential participants were then telephoned by a research team member to receive verbal information about the study with the opportunity to ask questions. If interest persisted, written participant information, a consent form, and a self-administered sociodemographic questionnaire (covering age, gender, employment, geographic location, time since cardiac arrest, and use of digital devices and internet habits) were mailed to the home address. Completed documents were returned in a prepaid envelope. Once consent was confirmed, participants were contacted to indicate their preference for partaking in a focus group or an individual interview. Offering both focus groups and individual interviews ensured that survivors who wished to share their experiences but were uncomfortable participating in a group setting could still take part. This flexible approach enhanced inclusivity and enabled the collection of complementary forms of data.

### Participants

Purposive sampling was used to ensure that participants possessed characteristics and experiences relevant to the study aim. Cardiac arrest survivors were recruited through the National Peer Support Network for Cardiac Arrest Survivors and Their Family Members. To obtain a broad range of perspectives, efforts were made to include variation in age, gender, time since the cardiac arrest event, arrest location (in- or out-of-hospital), and geographical region.

Inclusion criteria were (1)participants aged 18 years and older, (2) ability to speak and write Swedish, and (3) survival of a cardiac arrest at least 1 month prior to the interview. These criteria reflected the intended target group for the future program (adults and Swedish-speaking users) and ensured that participants were no longer in the most acute postevent phase. While participants were included according to these criteria, diversity in background characteristics was actively sought.

### Data Collection

Data were collected through 4 focus group interviews (4‐5 participants per group) and 3 individual interviews. The sample size was guided by the principle of information power to ensure that it was large and varied enough to elucidate the study aim [29]. The total number of interview sessions and participants was determined in a stepwise manner. Sufficient information power was considered to have been achieved, given the study’s narrow aim, the high specificity of the sample, the strong quality of the interview dialogue, and a combined analytical strategy partly focused on in-depth experiential exploration.

The focus groups were conducted in accordance with Krueger and Casey’s [30] recommendations regarding group composition, question design, and moderation techniques, while the individual interviews followed Kvale and Brinkmann’s [31] principles for qualitative interviewing, emphasizing openness, depth, and reflective listening. The face-to-face focus groups took place in a hospital consultation room and at the Swedish Heart and Lung Association, and the virtual groups were conducted via Zoom (Zoom Communications, Inc). The individual interviews were held at locations chosen by the participants, either in their home or in a hospital consultation room.

Before each interview, the interviewer clarified that the purpose was to explore survivors’ perspectives on digital support as a complement to existing health care services, and participants were given the opportunity to ask questions. Both focus groups and individual interviews were guided by a semistructured, pilot-tested interview guide containing open-ended exploratory questions, followed by reflective prompts when appropriate (Textbox 1).

Textbox 1. **Interview guide.**

Questions:

Before the interview began, the interviewer explained their reasons for, and interest in, the research topic.

Introductory questions

- Could you start by describing your experience following the cardiac arrest and how you managed daily life (eg, what was most challenging or helpful)?
- What kind of support did you receive from those around you (eg, family, friends, and social networks)?

Support from health care

- Can you describe the support you received from health care services after the cardiac arrest?
- How do you perceive the need for support from health care in the aftermath?
- What forms of support do you think would be appropriate (eg, phone calls and in-person meetings—group or individual)?

Educational needs

- What information or guidance did you receive from health care after the cardiac arrest?
- How do you view the need for further information or education in this context?
- In what format could such education be delivered?

Future digital support and education program

- What is your view on the potential for a digital support program, delivered via a website, to complement existing health care services?
- If relevant, what kind of content should such a program include?
- How should the content ideally be presented?

Closing reflections

- Finally, is there anything we haven’t discussed that you believe is important to highlight?

Follow-up prompts

- Probing questions such as “Could you tell me more?” “What do you think about that?” or “Have I understood you correctly?” were used to deepen or clarify responses.

The focus groups were moderated by the second author (AB), an ambulance nurse and researcher with extensive experience of qualitative data collection and clinical experience in supporting survivors and their families. The first author (AW), who is a cardiac specialist nurse and researcher with expertise in post–cardiac arrest care, attended as an observer, responsible for taking notes, documenting observations, and posing supplementary questions when appropriate. The individual interviews were conducted by AW using the same semistructured guide.

**Figure 1.**
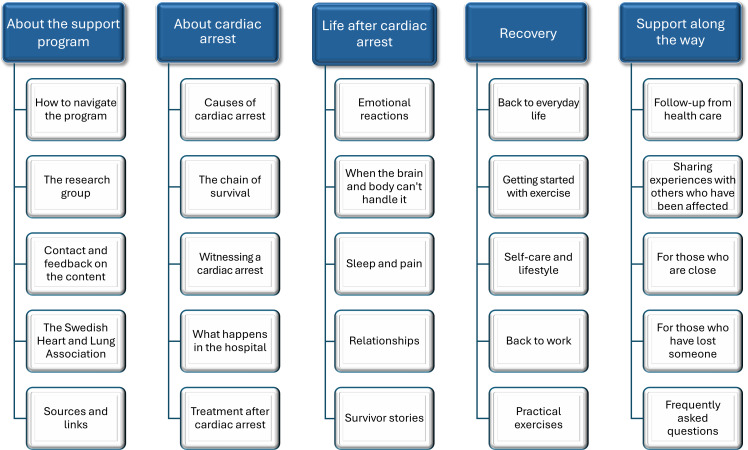
Preliminary paper-based schematic overview used to facilitate discussion about the content and structure of a future co-designed web-based support and learning platform.

Participants were encouraged to speak freely, share recovery experiences and perceptions in their own words, and reflect on what aspects of support and content a complementary digital format could provide beyond existing information sources. An open, dialogic atmosphere was fostered through active listening. After participants had first reflected freely on the potential for a digital support and education program and discussed what such a program might include, they were shown a preliminary paper-based schematic overview illustrating suggested content areas and structural components (Figure 1). The overview, developed by the research team and informed by a review of existing peer-driven platforms, such as Heartsight and Sudden Cardiac Arrest UK, was presented as a provisional discussion tool. Participants were informed that its purpose was to elicit feedback rather than to propose a fixed solution. They were invited to comment on the content, suggest additions or modifications, and reflect on the overall structure, including the use of overarching headings with clickable subtopics.

All interviews were audio-recorded and transcribed verbatim by AW. The focus group interviews lasted 96‐113 minutes and the individual interviews lasted 27‐44 minutes. In total, the dataset comprised 55,901 words, 835 citations, and 98 single-spaced pages.

### Data Analysis

Qualitative content analysis was used to systematically summarize the data while preserving their informational value and contextual depth [32]. The same analytic approach was applied to both focus group and individual interview data. This enabled the identification of recurring categories, patterns, and contrasts that contributed to a nuanced understanding of participants’ perceptions and proposals.

According to Krippendorff [33], qualitative content analysis is a systematic method for describing and interpreting verbal communication, with emphasis on contextual meaning rather than on quantification. While some approaches of content analysis may include attention to frequencies, Krippendorff highlights that the primary aim is to generate meaningful interpretations through a transparent and consistent analytic process. This involves systematically developing and applying a coding scheme across the dataset so that the analytic decisions can be followed and scrutinized by other researchers, thereby supporting reliability and validity. Meaning is understood in relation to its broader context, requiring the researcher to attend to both content and context when interpreting the data. The coding process is iterative, with categories being developed, refined, and sometimes merged or split as the analysis progresses, ultimately revealing patterns and interpretations that help explain the data [33].

The initial coding and analysis were conducted by the first author (AW), following a thorough and structured process described by Graneheim and Lundman [32]. First, all transcripts were read repeatedly to obtain a sense of the whole, with attention paid to both diversity and commonalities in participants’ experiences, perceptions, and proposals. The text was then divided into coding units, which were subsequently condensed while retaining their core meaning. These units were further labeled with codes. Finally, the codes were grouped into subcategories and main categories to represent the manifest content (Table 1). Emerging interpretations (latent content), subcategories, and main categories were then iteratively discussed with the coauthors to ensure credibility and agreement between authors.

**Table 1.** Example of the analysis.

Quote	Coding units	Code	Subcategory	Main category
“Not too long texts on what you see first, then you should be able to delve deeper, I think. But long, really long factual texts are really hard for me. It’s hard to read. Brief information should come first. It will make it easier for those with acquired brain damage if they have aphasia or something like that and have it read out to them.”	Not too long texts. Be able to delve deeper. Very long factual texts are hard to read. Brief information should come first. Make it easier for those with acquired brain damage if they have aphasia to have it read to them.	Short texts are preferable and should come first. Longer factual texts should be available for those who want to read more. Read-out texts help with cognitive impairment.	Short, easy-to-read texts with audio option.	Clear and tailored digital communication.
“I also think it would be smart if the support program was in an app, since more and more people are using mobile phones and tablets instead of computers. It doesn't have to be an advanced app, but it would be important that the function is available there.”	Smart if the support program was in an app. People are using mobile phones and tablets instead of computers. It doesn't have to be an advanced app.	The support program must be available as an app. People use phones and tablets rather than computers.	The support program should be offered as an app.	Easier access by an app.

### Reflexivity and Positionality

As this study constituted the initial step in a broader co-design process, the researchers were actively engaged throughout data collection and analysis. This involvement, together with the team’s clinical and academic experience in post–cardiac arrest care, provided valuable contextual understanding but also introduced a potential risk of preunderstanding shaping the interpretation. To address this, the researchers engaged in continuous reflexive dialogue, critically considering how their prior preunderstanding and commitment to the development process might influence analytic decisions. Coding and emerging interpretations were discussed iteratively to ensure that the analysis remained grounded in participants’ accounts rather than researcher assumptions.

### Ethical Considerations

The study complies with the principles outlined in the Declaration of Helsinki and received approval from the Swedish Ethical Review Authority (reference number 2024-01840-01). Prior to the focus group sessions, all participants were asked to agree to a confidentiality agreement, ensuring that all discussions remained within the group and that everyone would have an equal opportunity to share their experiences. Participants received both written and verbal information about the study’s purpose, the voluntary nature of participation, and their right to withdraw at any time. Written informed consent was obtained from all participants prior to participation in interviews or focus groups. Participant confidentiality and privacy were strictly maintained throughout the study. All participants were assigned pseudonyms, and all interview data were anonymized during transcription. Any potentially identifying information was removed or altered to protect confidentiality. Audio recordings and transcripts were stored securely on password-protected computers accessible only to the research team. The study included participants who had experienced cardiac arrest over an 18-year period, which further reduces the risk of individual identification. Nevertheless, care was taken to ensure that no information presented in the findings could reasonably lead to identification of participants. Data on computer, mobile phone, and internet use, as well as cardiac arrest–specific variables including years since cardiac arrest, age, implantable cardioverter-defibrillator (ICD), location of cardiac arrest, etiology, and post–cardiac arrest follow-up, were collected individually using written questionnaires before the interviews. All questionnaires were coded, and the code list was stored separately in a locked cabinet at the research unit. Access to both the data and the code list was restricted to a single member of the research team in order to ensure participant privacy and confidentiality. No financial compensation was provided. The research team recognized that the interviews could evoke distressing memories or suppressed emotions related to the cardiac arrest event. To support participants, contact details for a designated member of the research team were provided, in case further support was needed. The research team has extensive clinical and research experience working with this patient population, which contributed to ethical sensitivity throughout the study process.

## Results

### Participant Characteristics

A total of 20 participants took part in the study. Participants ranged in age from 44 to 80 years and included 8 women and 12 men. Time since cardiac arrest varied from 3 months to 19 years. Twelve participants had received an ICD following their cardiac arrest. The sample represented broad geographical diversity, with participants residing in southern, central, and northern Sweden. Myocardial infarction was the most commonly reported underlying cause of the cardiac arrest, particularly among men (Table 2). All participants reported daily use of both a computer and a smartphone, and 19 participants used the internet daily. Six participants also used a tablet.

**Table 2.** Background characteristics.

Characteristics	Women (n=8)	Men (n=12)	Total (N=20)
Years since cardiac arrest, median (range)	2.5 (0‐8)	7.0 (3-19)	3.5 (0‐19)
Age (years), median (range)	53.0 (44-63)	67.0 (46-80)	62.5 (44-80)
ICD^a^ recipient (yes), n (%)	4 (50)	8 (67)	12 (60)
Location of cardiac arrest, n (%)			
At home	4 (50)	3 (25)	7 (35)
In a public place	3 (37.5)	9 (75)	12 (60)
In hospital	1 (12.5)	—^b^	1 (5)
Etiology of cardiac arrest, n (%)			
Myocardial infarction	2 (25)	10 (83)	12 (60)
Arrhythmia	1 (12.5)	1 (8.5)	2 (10)
Cardiomyopathy	1 (12.5)	—	1 (5)
Pulmonary embolism	1 (12.5)	—	1 (5)
Other	3 (37.5)	1 (8.5)	4 (20)
Received hospital follow-up (yes), n (%)	6 (75)	11 (92)	17 (85)

a ICD: implantable cardioverter-defibrillator.

b Not available.

### Categories and Subcategories

**Figure 2.**
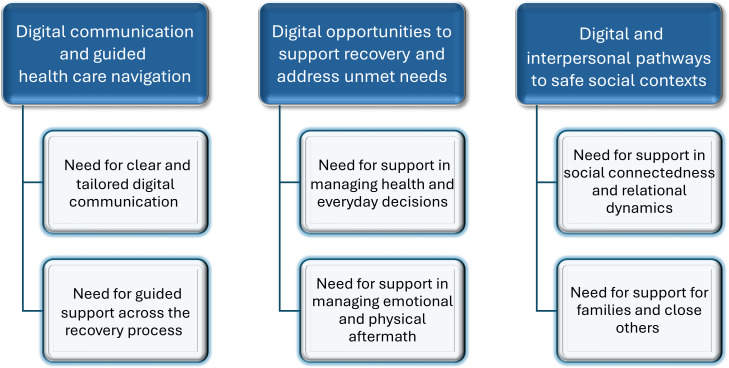
Conceptual overview of 3 core unmet needs identified from cardiac arrest survivors’ interviews (Waldemar et al [11]). The categorization reflects survivors’ views of digital support as a complement to existing care structures. The figure illustrates survivors’ calls for (1) digital communication and guided health care navigation, (2) digital opportunities to support recovery and address unmet needs, and (3) digital and interpersonal pathways to safe social contexts.

The results illustrate survivors’ preferences regarding the design, content, and delivery of user-friendly digital support. These preferences were grounded in their lived experiences of recovery, which shaped their expectations of what a digital program should provide. In describing these experiences, participants also reflected on how a digital support resource could address the needs they identified. The findings are presented in 3 main categories and 6 subcategories (Figure 2 [11]).

### Digital Communication and Guided Health Care Navigation

Dispersed and sometimes contradictory information from different health sources created a need for a digital program that integrated, clarified, and tailored guidance across the care continuum. Effective digital accessibility was understood to depend not only on technical design but also on usability, linguistic clarity, and cognitive manageability. Digital communication that was easy to navigate, easy to process, and available in multiple formats—such as links and videos—enhanced inclusivity by accommodating diverse user needs and capabilities. The centralization of credible and trustworthy resources within a single, intuitive interface was regarded as essential for reducing exposure to misinformation. Trust in such systems was shaped by the degree of transparency, clarity of information sources, and the presence of professional endorsement. Structured digital guidance was considered necessary across all phases of care—from the cardiac arrest event and prehospital interventions to acute hospital treatment, discharge, and long-term adjustment.

### Need for Clear and Tailored Digital Communication

A central aspect of participants’ vision for a digital support program was the need for communication that was cognitively accessible, emotionally attuned, and practically useful. Survivors described how difficulties with memory, concentration, and information processing made it challenging to engage with conventional health information, particularly when written in complex language or presented in fragmented ways. This often led to confusion and disengagement: “It all gets muddled in my head. I end up just reading headlines in the news—anything more and I lose focus.”

To address these challenges in a digital format, participants advocated for layered communication: short, plain language summaries with optional links to more detailed content. Concise, easily accessible, and multimodal information was consistently emphasized. Short texts were preferred, as extensive reading was perceived as overwhelming, and participants valued the option to access more in-depth material through linked pages. Detailed explanatory texts, including glossaries, were requested: “Hospital language is something you can't keep up with...nothing makes sense. So, a glossary would really help.” These features were seen not only as tools for understanding but also as signs of respect for the cognitive realities many face after cardiac arrest.

Multimodal formats were considered essential. Participants highlighted the importance of text-to-speech functionality and translations into widely spoken languages, such as English and Arabic, to enhance accessibility: “It would be great if the text could be read out loud as you go.” Visual communication was also emphasized, particularly for those experiencing cognitive fatigue. Participants appreciated having alternative formats, such as the option to engage with content through text or video. Short videos or read-aloud text were described as easier to interact with than traditional written material.

The inclusion of lived experience quotations was suggested as a way to foster recognition and emotional connection within the digital environment. Visual elements were viewed as important for supporting understanding, with a clear preference for photographs over stylized illustrations: “They used illustrated images at first and I couldn’t make sense of them. But when they switched to real photos, I understood immediately.”

Participants also envisioned the platform as a space for connection with trusted networks. Survivor narratives were considered valuable for reducing feelings of isolation and fostering recognition: “I just wanted to talk to someone who’d been through the same. Someone who gets it.” The inclusion of family members’ perspectives was also requested: “Families’ stories are important too—so they feel seen and recognised.”

### Need for Guided Support Across the Recovery Process

Participants emphasized the importance of including structured guidance throughout all stages of the recovery in the support program. A recurring wish was for step-by-step information that could clarify what had happened, explain what to expect, and provide guidance moving forward. Many survivors described the acute phase—particularly the hospital stay following an out-of-hospital cardiac arrest—as fragmented or entirely absent from memory. To support understanding of this phase, participants proposed a dedicated section or timeline within the program, tentatively titled “What happened at the hospital,” outlining the care trajectory from ambulance arrival to discharge. Emergency interventions such as cardiopulmonary resuscitation (CPR), defibrillation, intraosseous access, and mechanical chest compressions were frequently mentioned as sources of both physical pain and emotional confusion.

Experiences from the intensive care unit (ICU) were similarly described as surreal or frightening. Participants stressed the importance of including information in the digital program that could explain these experiences and normalize common reactions. “I was hallucinating after I woke up...Later I was told this is common after being in the ICU. But at the time, I thought I was going mad.”

The transition from hospital to home was identified as a particularly vulnerable phase, and participants called for this to be addressed explicitly in the program. While some described receiving adequate support, others reported being discharged without sufficient preparation or follow-up. “It’s incredibly valuable when someone from the hospital calls and asks, ‘Is there anything you need?’ Rather than just disconnecting you from the telemetry and leaving you to figure it out on your own.”

Follow-up care was experienced as inconsistent and dependent on regional practices. Participants emphasized that existing national guidelines for post–cardiac arrest care are not uniformly implemented and suggested that these should be clearly presented within the program. Access to cardiac rehabilitation was described as essential for regaining both confidence and physical strength. “When I was transferred to primary care, all care just stopped...I didn’t know who to turn to.” To address these gaps, participants proposed that the digital program should include a clearly structured pathway, outlining key stages and responsibilities across the recovery timeline. These suggestions reflected participants’ desire for a coherent and continuous source of guidance across the entire recovery journey.

### Digital Opportunities to Support Recovery and Address Unmet Needs

Challenges in managing the physical, cognitive, and emotional consequences of cardiac arrest highlighted the need for digital resources that provided comprehensive, long-term recovery support extending beyond standard medical follow-up. Recovery was characterized as a multifaceted process involving both clinical management and ongoing emotional adjustment. Digital support was required to improve understanding of causes, treatments, and medications, as well as to address managing cognitive and physical difficulties such as fatigue, pain, and memory impairment. Unmet needs related to psychological and existential concerns were evident, and limited access to counseling or psychological interventions underscored the importance of incorporating dedicated modules addressing these aspects of recovery. Clear, accessible, and adaptable guidance was required to support survivors in navigating the often-overlooked phase of postdischarge life in ways that current information pathways do not consistently provide. This need was best supported by a flexible, modular design capable of adjusting to varying needs, preferences, and recovery trajectories.

### Need for Support in Managing Health and Everyday Decisions

Participants were asked to reflect on how challenges could be supported digitally. They highlighted a range of topics that they believed should be addressed in a digital program to help navigate everyday decisions and uncertainties after cardiac arrest. Existing written and oral patient information was often described as insufficient, fragmented, or difficult to revisit, whereas a digital format could offer structured explanations, tailored guidance, and practical tools such as video-based exercises to support long-term recovery. Participants appreciated the idea of having all relevant information gathered in one place, presented in an accessible way, and available both as concise and more in-depth text:

*During the first year, I searched for an enormous amount of information. I wanted to hear other people’s stories, see their experiences. I listened to some British podcast that I thought was good at the time, and I read a lot of scientific papers—but that’s a language you just can’t follow. And these general brochures for us ordinary folks...eventually you’ve read all of them, and then you end up with the medical papers instead. And you’re really interested and curious because it feels spot-on for you, but you don’t understand a thing*.

A central concern was the lack of clarity regarding the cause of the arrest. This uncertainty was described as difficult to live with and generated a strong desire for accessible on-demand information in a consolidated space with explanations of possible underlying mechanisms. “I wish it had been something with my heart they could fix, or that the ICD made me feel safe. But they don’t even know why I had the arrest.” Some participants described confusing cardiac arrest with myocardial infarction and emphasized the importance of having key medical concepts explained in clear, plain language within the program.

Understanding prescribed medication was another area requiring targeted support centralized within 1 trustworthy digital resource. Survivors often felt underinformed about the purpose of their medications and were hesitant to seek clarification online due to fear of misinformation or unnecessary anxiety. “Why am I taking beta-blockers? I still don’t know...I don’t want to Google too much because then you think you have every side effect.” Survivors also described discontinuing medications because of side effects or unclear instructions. In response, participants proposed that the digital program should offer personalized, trustworthy information about medications and their role in secondary prevention.

Living with an ICD was another example where participants expressed a strong desire for digital structured information and emotional guidance, since existing health resources were experienced as insufficient, diffuse, and sometimes contradictory. Similarly, survivors who had undergone interventions following myocardial infarction requested clearer explanations of what had been done and what the procedures meant for their long-term health.

Cognitive impairments were described as a prominent and often unexpected consequence, with longer-lasting effects than many survivors had anticipated. Acceptance of these changes was reported to take time. Participants therefore recommended that the program should include practical cognitive rehabilitation exercises, such as memory training tools, occupational therapy, and guidance on reintegration into work and everyday activities. “In the beginning, it was really hard to accept help...But the rehab has been incredibly helpful.”

Preventing recurrence and regaining a sense of control were described as unmet needs that were particularly suited to digital delivery, especially when expert-based information with links for further reading and practical exercises could be accessed from home in a calm and familiar setting. Participants requested practical, evidence-based advice within the program on everyday concerns, including diet, alcohol, physical activity, sexual health, and sleep. Exercise, in particular, was described as anxiety-provoking, with many survivors uncertain about what levels of exertion were safe. “My brain doesn’t want to push too hard...I asked for an extra exercise test...Now I know I can get my heart rate up to 165 without it being dangerous.”

### Need for Support in Managing Emotional and Physical Aftermath

In addition to medical and practical concerns, participants highlighted the need for the digital support program to address the emotional and embodied consequences of surviving a cardiac arrest. Many described persistent physical sensations—such as chest discomfort, palpitations, and breathlessness—that triggered fear and uncertainty. As one participant asked: “If I get pressure on my chest, should I go to the emergency department? I’ve no idea.” To meet this need, participants expressed a strong desire for access to digital resources and interventions to support symptom management and receive guidance on interpreting bodily signals and recognition when medical attention is warranted. Pain from CPR-related injuries, such as rib fractures, further compounded this distress. One participant recalled: “I was in so much pain, and everyone just laughed. Just a smile—‘at least you’re alive.’”

Sleep disturbances emerged as another significant and underaddressed issue in existing written information. Participants described racing thoughts at bedtime, fear of dying in their sleep, panic attacks during the night, and recurring nightmares. These experiences highlighted a need for video-based exercises and strategies to improve sleep, manage nocturnal anxiety, and recognize trauma-related symptoms.

Cognitive impairments, including memory problems, fatigue, and what participants referred to as “brain fog,” were among the most disruptive aspects of recovery. Many reported struggling with concentration, “zooming out,” or falling asleep during conversations or while sitting upright. Participants stressed that the program should explain the neurological effects of cardiac arrest and provide realistic information about recovery trajectories, including the slow and often unpredictable nature of cognitive rehabilitation. To accommodate difficulties with concentration and fatigue, participants emphasized the importance of offering this material not only in written form but also as short, video-based content for days when reading felt too demanding. As one participant noted: “I thought I’d be back at work by December...there’s no way. I’m exhausted.”

Existential fear and emotional upheaval were also prominent themes, which participants felt warranted a dedicated module within the program. Survivors described profound shifts in priorities, relationships, and identity. While some expressed gratitude for survival, others lived with a persistent fear of recurrence that shaped everyday life. One participant reflected: “My child now says ‘goodbye’ instead of ‘goodnight.’ That’s how scared we are.” Several participants noted that these fears were often minimized or dismissed by health care providers. “My doctor said, ‘But it’s been quite a while since your arrest,’ even though I’d had a second one two years later.”

Participants emphasized that the program should provide clear information about available psychological support services and offer guidance on how to access appropriate help when needed. The need to talk openly about these emotional and existential experiences was described as both urgent and unmet. Participants expressed frustration that family members, friends, and even clinicians often avoided such conversations. Psychological interventions and cardiac-specific counseling were highlighted as crucial yet inconsistently available resources, with most participants reporting that they had received no psychological follow-up.

### Digital and Interpersonal Pathways to Safe Social Contexts

Relational and emotional dynamics following cardiac arrest highlighted the need for a digital program that normalized common reactions, supported family communication, and provided guidance on intimacy, safety, and social reintegration. Cardiac arrest often disrupted established relationship patterns and generated fears related to solitude and vulnerability, underscoring the importance of connection, closeness, and relational stability. The broader social context required mechanisms that addressed intimacy, everyday interactions, and the reestablishment of a sense of interpersonal safety.

Family members emerged as essential sources of support, yet they were also placed in vulnerable positions and were frequently overlooked within formal recovery structures. These dynamics indicated a need for a digital resource that offered counseling, practical guidance, and tools to help families cope with emotional strain, monitor health-related changes, and navigate the challenges of daily life. A digital support needed to incorporate clear information, practical caregiving strategies, and validation of family experiences, ensuring that family involvement was recognized as an essential component of survivor recovery.

### Need for Support in Social Connectedness and Relational Dynamics

Participants discussed how a digital resource could help normalize relational experiences and provide guidance that could help navigate the social consequences of cardiac arrest that many described. Participants emphasized that the digital support program should address the profound social and relational consequences of surviving a cardiac arrest. The event was described as a turning point that disrupted identity and altered relationships, both with the self and with others. Many reported feeling like a different person after the arrest, often struggling with changes in personality or self-image: “It’s really strange in my head. Who am I now? I’m not myself anymore—such a huge part of my personality has been taken from me.” These internal changes were frequently reflected in how survivors were perceived and treated by others. Well-meaning but overprotective responses from family and friends could feel diminishing: “It feels like everyone looks down on me, even my children—who actually need their mother. That part is quite hard.”

Participants stressed that the program should include information about how cardiac arrest can affect social roles and offer practical strategies for navigating altered relational dynamics. Parent-child relationships were often reshaped, with children—both young and adult—assuming new responsibilities or roles. The program was seen as a potential digital space for sharing strategies to support families in understanding and negotiating each other’s ways of coping.

A recurring concern was the fear of being alone, both physically and emotionally. Survivors described intense feelings of isolation, exacerbated by the rarity of their experience and uncertainty about recurrence: “My first thought when I woke up was—I can’t be alone in this. But I felt very alone.” Participants suggested that the program could help normalize these reactions and provide guidance on how to seek emotional and practical support when needed. In addition to helping survivors cope with these stressors, digital peer support was noted as a means of supporting social connectedness for both survivors and close family.

Many also described social withdrawal or distancing by friends and colleagues, which they attributed to others’ uncertainty about how to act or respond. This was experienced as deeply painful and, in some cases, led to a profound sense of abandonment. Several participants also reported the breakdown of romantic relationships following the arrest, frequently related to emotional strain or unresolved trauma: “My boyfriend couldn’t handle it—it ended there. So now I feel very alone.”

Sexuality and intimacy were additional areas participants felt must be explicitly addressed in the program since these were lacking in existing written information. Survivors described how cardiovascular medications negatively affected sexual desire and function, issues rarely acknowledged in clinical encounters: “Beta-blockers and blood pressure meds are no party boosters...It would’ve been much easier if someone had just said: Things might not work so well in bed.” Participants emphasized that existing support was fragmented, difficult to access, or entirely absent, whereas a digital format could offer normalizing information, family-oriented guidance, and practical tools to strengthen communication, intimacy, and social reintegration. In this way, participants viewed a digital program as a means to create safer social contexts and to support the relational dimensions of recovery that are often overlooked in standard care.

Many worried that sexual activity might trigger another cardiac arrest, particularly in relation to arrhythmias, but felt too embarrassed or uncertain to raise these concerns. When such topics were eventually discussed, responses were often reassuring, underscoring the need for the program to provide clear, proactive, and destigmatizing information about sexual health and intimacy after cardiac arrest. Overall, participants underscored that rebuilding meaningful social connections and addressing intimacy concerns were essential components of recovery and should be treated as such within the digital support program.

### Need for Support for Families and Close Others

Participants highlighted the need for a dedicated section in the digital support program for family members, describing them as both central to the cardiac arrest experience and profoundly affected by it. Despite their crucial role, family members were often perceived as overlooked by health care professionals: “There’s a lot of focus on the one who survived...but someone else was there too, watching over you in those first two days.” Because family members rarely receive formal follow-up or information tailored to their specific needs, participants viewed a digital format as particularly suitable for providing accessible, reliable, and continuous support.

Survivors recounted how traumatizing the cardiac arrest had been for their loved ones—sometimes more than for themselves. Many family members received abrupt phone calls, arriving at the hospital to find the survivor sedated and unresponsive: “We said goodbye in the morning, and then they get a phone call saying I’ve had a cardiac arrest.” Witnessing CPR, waiting through prolonged transfers, or being separated during emergencies contributed to a pervasive sense of helplessness and lasting trauma. Participants therefore viewed a program module explaining early care trajectories and common reactions among family members as particularly valuable during the acute phase. As such content is largely absent from existing written materials, a digital platform available 24/7 was considered an important way to provide structured explanations and reassurance during this initial period.

Children and adolescents were described as especially vulnerable yet rarely offered appropriate support. Some had performed CPR or witnessed the arrest and subsequently struggled with long-term fear and anxiety: “My husband and son, who were fourteen at the time, did CPR. The kids didn’t cope very well...they were frightened for a long time.” Attempts to access psychological support for children were often unsuccessful. Children and grandchildren were deeply affected by the event, sometimes developing separation anxiety or persistent worry about the survivor’s safety. Participants reported that they did not fully recognize the extent of their loved ones’ trauma until much later and therefore emphasized the need for age-appropriate guidance and tools for supporting children and young people. A digital program was viewed as a practical way to provide families with accessible support when professional services were unavailable.

Partners and close family members were also described as being physically affected by the emotional toll, developing stress-related illness or severe exhaustion. Participants clearly articulated that when the needs of family members are overlooked, this has a direct impact on their own recovery: “Support for the family is also support for us, the survivors.” As family members often carry this responsibility without preparation or guidance, participants saw digital support as a way to provide timely information and reassurance in situations where health care contact was limited.

Following hospital discharge, family members frequently assumed extensive caregiving responsibilities without preparation or guidance. They were expected to monitor symptoms, interpret medical information, and make decisions amid uncertainty. Vigilant behaviors—such as checking breathing at night or using apps to track the survivor’s location, activity, or heart rhythm—were common and driven by the family’s own fear of recurrence. Cognitive impairments, fatigue, and emotional changes in the survivor further intensified this burden.

Friends and bystanders who had performed CPR or witnessed the event were likewise described as emotionally affected but received no follow-up, debriefing, or support. Digital peer support was also viewed as uniquely suited to these groups, as informal networks are otherwise difficult to access and no formal follow-up is routinely offered: “It must have been terrifying. They should have been offered help, but nobody contacted them.”

## Discussion

### Principal Findings

This paper explored cardiac arrest survivors’ perspectives on the essential content and structure of a web-based support and learning platform. Survivors described a need for digital resources that are tailored, cognitively accessible, emotionally attuned, and integrated with existing health care services. While many of the cardiac arrest experiences identified have been reported previously, our findings extend this knowledge by demonstrating how these experiences translate into concrete design requirements for a digital support program. These insights represent the initial phase of a multiphase development process to cocreate, design, and later evaluate a web-based support and learning platform for cardiac arrest survivors.

Existing community-based and online resources for cardiac arrest survivors demonstrate the value of peer-driven information and shared lived experiences. Recent international surveys also show that such organizations play an important role in addressing unmet informational and emotional needs and yet remain only loosely connected to formal health care systems. Participants in our study described needs that extend beyond what these initiatives currently offer, including structured, clinically endorsed, and contextually adapted digital support. Survivors highlighted the importance of trustworthy information, guidance across care transitions, and content aligned with their cognitive, emotional, and practical recovery needs. Our findings therefore contribute to user-driven design requirements that can inform the development of a more comprehensive, evidence-informed digital support program tailored to the Swedish health care context.

### Comparison With Prior Work

Participants’ difficulties with memory, concentration, and information processing align with previous research on health literacy and digital engagement among individuals with cognitive impairments [34,35]. Their preferences for layered, plain language communication are consistent with principles of universal design [36]. Requests for visual aids, text-to-speech functionality, and multilingual options echo recommendations in the WHO Global Strategy on Digital Health 2020‐2025 [37] and reinforce the importance of inclusive digital tools that accommodate cognitive diversity [38,39].

Survivors’ memory gaps during the acute phase, particularly in the ICU, are well documented [3,40,41]. Our findings extend this work by showing how these gaps translate into a specific digital need: a clear, structured timeline explaining what happened; why certain procedures were performed; and what to expect next. Such features may support meaning-making, reduce distress, and enhance trust in the recovery process.

Participants’ desire for step-by-step guidance across the recovery trajectory resonates with the call for rigorous interventional studies addressing survivor- and family-focused support by Douma et al [42]. Transfers between care settings were described as emotionally significant, echoing earlier work showing that families often experience the ICU as both traumatic and reassuring, with distress increasing during transitions [43]. Digital support may help bridge these gaps by providing consistent information, clarifying roles, and outlining available services.

The cognitive and emotional aftermath of cardiac arrest—including fatigue, memory loss, anxiety, and existential distress—has been widely reported [44-46]. Cognitive impairment, depression, and reduced mobility have been linked to lower societal participation [47]. Our findings add to this literature by identifying digital features survivors believe would support recovery, such as modules explaining neurological recovery, strategies for managing fatigue, and guidance on when to seek medical attention. Participants’ reports of feeling dismissed by health care providers underscore the need for digital content that normalizes common symptoms, sets realistic expectations, and offers psychological coping strategies.

Survey data from the Danish Cardiac Arrest Survivors study further highlight the long-term nature of these challenges, with many survivors reporting persistent symptoms and incomplete recovery up to 5 years postevent [48]. Similarly, Mion et al [7] found that survivors and families prefer early follow-up within the first month. These findings reinforce the need for digital tools that provide timely, realistic, and ongoing support.

Survivors’ descriptions of altered identity, family role shifts, and challenges related to intimacy and social reintegration align with research on traumatic brain injury [49,50] and studies showing that living with an ICD can contribute to marital strain [43]. Our study extends this work by demonstrating how these relational challenges translate into digital needs, including content on communication within families, guidance on intimacy and sexuality, and peer stories that normalize emotional reactions. Southern et al [43] similarly describe the postarrest period as one of existential reevaluation and renegotiation of roles, suggesting that digital support may help survivors and families navigate these transitions through psychoeducation and coping strategies.

Participants frequently described their family members as overlooked in the recovery process, echoing Haywood and Dainty’s [51] description of family members as “forgotten patients.” Evidence that survivors and their family members can influence each other’s health outcomes and recovery trajectories [52] and calls for family-centered care in cardiac arrest survivorship [53] further support the need for dedicated family-oriented content. A recent scoping review by Douma et al [42] identified 5 key domains of family support: attention during the cardiac arrest, collaboration with the resuscitation team, contextual understanding, postresuscitation needs, and implementation of family-centered care. These domains align closely with the needs identified by participants in our study. Emerging digital psychoeducational interventions targeting survivors and families [54] highlight growing interest in this area. Our findings suggest that family-centered content should be integrated as a core component of digital support programs rather than a supplementary component.

### Implications for Digital Intervention Development

Taken together, our findings indicate that digital support tools for cardiac arrest survivors should not only deliver medical information but also foster emotional connection, peer support, and family inclusion across care transitions. Grounding design in survivors’ experiences and preferences can help ensure that digital interventions are responsive and accessible. In this way, cocreating a digital support tool is not merely a technical endeavor but reflects survivors’ call for more consistent, equitable, and person-centered care after cardiac arrest. Such tools may complement emerging psychoeducational efforts and address persistent gaps in current care pathways, including the limited integration of existing community-based support organizations into formal health care systems. In doing so, this study addresses a critical gap in current survivorship research by specifying the user-driven requirements necessary for developing effective, scalable digital support.

### Strengths and Limitations

A key strength of this study lies in its grounding in the experiences of end users. By actively incorporating survivors’ perspectives in a cocreative process, the research aligns with person-centered principles and enhances the relevance and applicability of its findings to real-world care and support systems.

Purposive sampling enabled the inclusion of participants with diverse experiences of cardiac arrest, capturing variation in underlying etiologies, age, time since the event, and geographic location. Participants had been hospitalized in different health care settings and received varying degrees of follow-up, which strengthened the contextual richness and transferability of the findings. However, recruitment through peer networks may have favored individuals with higher engagement or digital literacy, potentially limiting the diversity of perspectives.

Focus group interviews generated rich data through participant interaction, complemented by individual interviews that offered flexibility and inclusiveness. Smaller focus groups facilitated open discussions, including existential reflections, and digital interviews enabled participation from across Sweden. This combination provided both breadth and depth of insight [55].

The research team’s clinical and academic background in post–cardiac arrest care may have shaped interpretive sensitivity. Although reflexive practice was used to minimize bias, researcher preunderstanding may still have influenced interpretation. Although generalizability is not the goal of qualitative research, the findings are likely transferable to similar populations outside Sweden. While individual recovery experiences and support needs are inherently personal, the study contributes knowledge that can guide the development of tailored interventions.

### Conclusions

This study demonstrates how cardiac arrest survivors’ multifaceted recovery experiences can be translated into actionable user-driven design requirements for a web-based support program. The findings indicate a clear need for digital support that extends beyond clinical encounters by addressing persistent gaps in follow-up, providing accessible and cognitively manageable information, offering dedicated resources for family members, and delivering stepwise guidance across care transitions. A coherent digital format—combining concise and in-depth text, short videos, read-aloud functions, and practical tools—emerged as central to accommodating fatigue, cognitive difficulties, and the need to revisit information throughout recovery. These insights outline key priorities for a clinically grounded and contextually adaptable web-based support program positioned to strengthen long-term recovery and everyday functioning for cardiac arrest survivors, forming the foundation for cocreation and iterative development within this multiphase project.

## Supplementary material

10.2196/84432 Checklist 1 COREQ (Consolidated Criteria for Reporting Qualitative Research) checklist.

## Abbreviations

COREQ: Consolidated Criteria for Reporting Qualitative Research
CPR: cardiopulmonary resuscitation
ICD: implantable cardioverter-defibrillator
ICU: intensive care unit
